# Surface Modification of PHBV Fibrous Scaffold via Lithium Borohydride Reduction

**DOI:** 10.3390/ma15217494

**Published:** 2022-10-25

**Authors:** Paweł Chaber, Grzegorz Tylko, Jakub Włodarczyk, Paweł Nitschke, Anna Hercog, Sebastian Jurczyk, Jakub Rech, Jerzy Kubacki, Grażyna Adamus

**Affiliations:** 1 Centre of Polymer and Carbon Materials, Polish Academy of Sciences, M. Curie-Skłodowska 34, 41-819 Zabrze, Poland; 2 Department of Cell Biology and Imaging, Institute of Zoology and Biomedical Research, Faculty of Biology, Jagiellonian University, Gronostajowa 9, 30-387 Kraków, Poland; 3 Institute for Engineering of Polymer Materials and Dyes, Łukasiewicz Research Network, Marii Skłodowskiej-Curie 55, 87-100 Toruń, Poland; 4 Department of Biotechnology and Genetic Engineering, Faculty of Pharmaceutical Sciences in Sosnowiec, Medical University of Silesia, Poniatowskiego 15, 40-055 Katowice, Poland; 5 Faculty of Science and Technology, Institute of Physics, University of Silesia in Katowice, 75 Pułku Piechoty 1, 41-500 Chorzów, Poland

**Keywords:** PHAs, PHBV scaffolds, surface modification, borohydride reduction, electrospinning

## Abstract

In this study, lithium borohydride (LiBH_4_) reduction was used to modify the surface chemistry of poly(3-hydroxybutyrate-*co*-3-hydroxyvalerate) (PHBV) fibers. Although the most common reaction employed in the surface treatment of polyester materials is hydrolysis, it is not suitable for fiber modification of bacterial polyesters, which are highly resistant to this type of reaction. The use of LiBH_4_ allowed the formation of surface hydroxyl groups under very mild conditions, which was crucial for maintaining the fibers’ integrity. The presence of these groups resulted in a noticeable improvement in the surface hydrophilicity of PHBV, as revealed by contact angle measurements. After the treatment with a LiBH_4_ solution, the electrospun PHBV fibrous mat had a significantly greater number of viable osteoblast-like cells (SaOS-2 cell line) than the untreated mat. Moreover, the results of the cell proliferation measurements correlated well with the observed cell morphology. The most flattened SaOS-2 cells were found on the surface that supported the best cell attachment. Most importantly, the results of our study indicated that the degree of surface modification could be controlled by changing the degradation time and concentration of the borohydride solution. This was of great importance since it allowed optimization of the surface properties to achieve the highest cell-proliferation capacity.

## 1. Introduction

Fibrous polymeric materials have been widely used for tissue engineering purposes [1,2,3]. The primary goal of tissue engineering is to restore the volume and function of damaged or injured tissues [4]. Depending upon the type of tissue, various scaffold architectures with different morphological features can be used [5]. Among them, fibrous surfaces seem to have an advantage over flat ones because they nicely resemble the structure of the native extracellular matrix (ECM) [6,7,8,9]. One of the most versatile and straightforward methods that allow for obtaining biomimetic polymeric fibers is electrospinning [2,7,10]. Electrospun fibrous materials have been confirmed to be good candidates for ECM-mimicking scaffolds [8,9].

Polyhydroxyalkanoates (PHAs), due to their widespread presence in living organisms, have met with great interest in the field of biomedical sciences [11,12,13,14]. They are biocompatible and biodegradable linear polyesters, which degrade into non-toxic products [13,15]. These properties are of significant importance since it is expected that the implanted scaffold will not cause an inflammatory response and will be removed from the body after fulfilling its purpose. To better meet the potential needs of tissue engineers, the chemical composition of PHAs, and thus their physicochemical properties, can be regulated to some extent at the level of their biosynthesis in bacteria. To date, over 160 different monomeric units have been incorporated into PHAs by varying fermentation conditions [12,16,17]. It is not surprising then that PHAs have been widely explored as candidate materials for the regeneration of various tissues, such as bone, cartilage, skin, and nerves [5,13,15,18,19,20].

PHAs surely possess the properties that make them very promising as scaffolds for tissue engineering. However, they are not free of drawbacks. The lack of biological cues that promote adequate cell–material interactions is the most important one [12,21,22,23]. Once the cells are brought into contact with the construct, they should adhere to its surface, proliferate, and migrate throughout its volume. However, the lack of specific functional groups on the material surface makes the cells unable to perform these activities [24,25,26]. This deficiency limits the utility of PHAs as a direct replacement for damaged or diseased tissues. Since the surface of the implanted scaffold is the first thing encountered by cells and biological fluids, an easy way to improve the biological performance of the material is to modify its surface properties. Consequently, numerous surface modification strategies have been developed and explored for tissue engineering applications [27,28,29,30,31].

Surface modification approaches are generally divided into two groups, namely physical and chemical methods [28,32,33]. In contrast to modifications based on physical phenomena (including plasma treatment), chemically modified surfaces exhibit excellent long-term stability [33,34,35]. Moreover, the newly introduced functional groups are useful sites for further modification and linkage with other species [28,36]. It is important to note that surface chemistry plays a crucial role in cell–polymer interactions [24,37,38,39,40]. Generally, the introduction of surface polar groups, such as hydroxyl, carboxyl, amino, and sulfonic acid groups, promotes better cell adhesion, migration, and proliferation [26]. However, one functional group may influence a specific cell function to a greater extent than another. Curtis et al. studied such effects using polystyrene templates that varied in surface chemistry [41]. The study revealed that BHK cells adhered better on the surface with hydroxyl groups than that with carboxyl or sulfur-containing groups. In another study, Keselowsky et al. showed that the adhesion strength of MC3T3-E1 osteoblast-like cells to fibronectin-coated surfaces also depended on the surface chemistry [25]. Once again, surface hydroxyl groups appeared to be the most appropriate for the cell adhesion process, showing an increased attachment capacity of the bone-forming cells in comparison to carboxyl, amino, and methyl groups. However, hydroxyl groups are not always the most effective ones in promoting cell adhesion [42]. In fact, there are no general rules regarding the extent of cell attachment and growth on different polymer surfaces. Nonetheless, methods that allow the introduction of well-defined surface functionalities, including the already-discussed hydroxyl groups, are of great importance in tissue engineering.

The surface treatment of PHAs has been the subject of numerous research efforts [23,27,36]. To our knowledge, there are only five reports in the literature describing modifications of PHAs that result in the formation of surface hydroxyl groups [36,43,44]. Of these, only two concern the creation of hydroxyl groups through wet chemical treatment—that is, through alkaline hydrolysis and aminolysis; others are about the use of radiation-induced polymerization to graft poly(hydroxyethyl methacrylate) (PHEMA) onto the surface of interest [45,46]. However, the grafting of PHEMA onto PHAs may reduce the rate of their biodegradation [46]. On the other hand, neither aminolysis nor hydrolysis has been used to produce surface-modified PHA fibers. Aminolysis and acid-catalyzed hydrolysis require prolonged heating at relatively high temperatures to degrade PHAs [47,48,49]. Such conditions are not suitable for the surface modification of fibers since they may negatively impact their morphological and mechanical properties. The use of harsh conditions may also result in the destruction of the fibrous structure [31]. The base-catalyzed hydrolysis of PHAs, in turn, is accompanied by undesirable cis-elimination reactions [47,50]. The only methods that have been used to modify PHA fibers are based on the already mentioned radiation-induced polymerization, which may be carried out under relatively mild conditions [23,51,52]. This reaction allows the introduction of amide and carboxylic acid functional groups on the scaffold surface. However, the formation of free radicals from highly crystalline PHAs in a heterogeneous reaction system presents some difficulties [46].

This paper presents a simple way to enrich polyester surfaces with hydroxyl groups. The higher density of these groups was attained by using lithium borohydride (LiBH_4_). In our previous study, this reducing agent permitted us to obtain low-molar-mass poly(3-hydroxybutyrate) (PHB) with hydroxyl terminal groups, which was confirmed by GPC, proton NMR, and ESI-MS experiments [53]. The oligomers, of course, were synthesized in a heterogeneous system, that is, by reducing high-molar-mass PHB in the form of a powder. The mild conditions of their synthesis inspired us to use LiBH_4_ for the surface modification of a polyester-based scaffold consisting of electrospun fibers. Poly(3-hydroxybutyrate-*co*-3-hydroxyvalerate) (PHBV), one of the most well-studied members of the PHA family, was used as the scaffold material. The topographical features of the modified materials were characterized by AFM and SEM techniques, whereas the change in the surface chemistry was monitored by FT-IR spectroscopy. The possibility of controlling the degree of surface functionalization was also examined. In order to determine whether the LiBH_4_-modified surface promotes better tissue formation than the unmodified one, the fiber materials were subjected to preliminary biological tests.

## 2. Materials and Methods

### 2.1. Materials

Poly(3-hydroxybutyrate-*co*-3-hydroxyvalerate) (PHBV) containing 11 mol% of valerate units, 1,1,1,3,3,3-hexafluoro-2-propanol (≥99%), a 0.5 M solution of LiBH_4_ in diethyl ether, and a 1.0 M solution of HCl in diethyl ether were purchased from Sigma-Aldrich, Steinheim, Germany. Diethyl ether (99.5%), ethanol (96.0%), and chloroform (98.5%) were obtained from Avantor Performance Materials Poland S.A., Gliwice, Poland. All reagents were used as received. The PHBV film was prepared from a chloroform solution (12.5 cm^3^, 10% *w*/*v*) by casting it onto a Teflon plate of 10 cm diameter. The casted film was then cut into small square pieces of dimensions 20 mm × 20 mm. These pieces were vacuum-dried for 24 h at room temperature and eventually modified through the use of the LiBH_4_ solution.

### 2.2. Fabrication of Electrospun PHBV Mats

The fibrous mat was obtained by electrospinning a 20% PHBV solution in 1,1,1,3,3,3-hexafluoro-2-propanol (HFIP). HFIP is widely used as a solvent in electrospinning because it has a low boiling point (58 °C) and a high dielectric constant (polarity) [54,55]. The solution was fed into the electrospinning machine (TL-Pro-BM, Tong Li Tech, Shenzhen, China), which was equipped with two high-voltage power supplies. The first one, generating a positive voltage potential of 13 kV, was applied to the spinneret in the form of a steel needle (size G20). The second one, generating a negative potential of −3.5 kV, was applied to the rotating collector (a steel cylinder with a diameter of 27 mm). The rotation speed was set to 500 rpm. The distance between the nozzle and the receiver was 19.5 cm. The polymer solution was dispensed from a polypropylene syringe using a Harvard Apparatus PHD Ultra 4400 infusion pump at a rate of 3 cm^3^/h. A total of 17 cm^3^ of the solution was used to produce the nonwoven material. The chamber of the electrospinning device allowed for the maintenance of ambient conditions, that is, a temperature and relative air humidity of 18 ± 1 °C and 63 ± 3%, respectively. Before being subjected to borohydride modification, the mat denoted as PHBV was removed from the collector plate and cut into small square pieces of dimensions 20 mm × 20 mm and a mass of about 0.1 g. The surface of these pieces was eventually modified. The thickness of the as-spun fiber mat was 0.40 ± 0.02 mm. Additionally, a PHBV solution in HFIP was electrospun to form a second fiber mat, which was denoted as the TPHBV mat. The second of the two prepared mats differed from the first one in terms of the average fiber diameter and the overall mat thickness. It consisted of fibers with an average fiber diameter of 0.74 ± 0.20 µm. The TPHBV mat had a thickness of 0.14 ± 0.03 mm. A 4-cm^2^ piece of this electrospun mat was modified by immersion for 20 min in a 0.005 M LiBH_4_ solution (see below for more details). The fibers thus obtained were denoted as TPHBV20. The mat was prepared only for the purpose of SEM imaging. Unless otherwise noted, all analysis and results correspond to the PHBV mat.

### 2.3. Preparation of LiBH_4_-Modified PHBV Fibrous Mats and Films

A wrist shaker (Conbest, Kraków, Poland) was used for mixing purposes. The rate of shaking used was approximately 400 vibrations per minute. The surface modification was performed as follows: the square-shaped piece of PHBV material (a fibrous mat or a film) with a side length of 2 cm was placed in a round-bottom flask, to which 10 cm^3^ of diethyl ether was added. After 10 min of shaking, 0.1 cm^3^ of the LiBH_4_ solution was added to the flask. Therefore, the final concentration of the borohydride reagent was 0.005 mol/dm^3^. The reaction mixture was then shaken for 15 min at room temperature. After decantation of the excess LiBH_4_ solution, the mat or film was washed one time with 10 cm^3^ of diethyl ether. The material was again immersed in 10 cm^3^ of diethyl ether and subsequently acidified with an ethereal solution of HCl. After 10 min of shaking, the HCl solution was decanted off, and the material was washed successively with ether and ethanol (to remove adsorbed LiCl). Finally, the LiBH_4_-modified PHBV material was dried at 37 °C for 40 h. The mat or film thus obtained was designated PHBV15. By changing the modification time, the samples designated PHBV5, PHBV10, and PHBV20 were obtained. For instance, the PHBV5 mat was obtained by immersing the PHBV mat in a 0.005 M LiBH_4_ solution for 5 min. The plus (+) and minus (−) sign were used to denote that the PHBV fiber mat or film was treated with a 0.0075 and 0.0025 M LiBH_4_ solution, respectively. For instance, the PHBV15+ samples were obtained through the use of a 0.0075 M LiBH_4_ solution for 15 min. All the reaction times and concentrations used together with the respective sample denotation are summarized in Table 1. The blank sample (PHBV0) was prepared by shaking together the PHBV material with 10 cm^3^ of diethyl ether for 20 min. It was followed by 10-min acidification with an ethereal solution of HCl, washing with ether and ethanol, and drying for 40 h. Similarly, the blank sample for the TPHBV mat was prepared and denoted as TPHBV0.

### 2.4. Characterization of LiBH_4_-Modified PHBV Materials

Both the blank sample and the LiBH_4_-modified materials were characterized by several methods. The average molar masses and molar mass distribution of PHBV that constituted the fibrous mat were determined with respect to polystyrene standards by gel permeation chromatography (GPC). The GPC system consisted of a Shodex SE-61 refractive index detector (Showa Denko Europe GmbH, Munich, Germany), a Viscotek VE 1122 solvent delivery system (Malvern, Worcestershire, UK), and a set of two PL-gel 5 μm mixed-C columns (Agilent Technologies, Church Stretton, England). All of the samples were analyzed at 35 °C. The elution solvent used was chloroform at a flow rate of 1 mL/minute. The samples were dissolved in chloroform at a concentration of 1% (*w*/*v*). The injection volume was 10 μL.

The morphology of the electrospun fibers was investigated using a scanning electron microscope (SEM, Quanta 250 FEG, FEI, Hillsboro, OR, USA). The samples were gold-coated prior to their analysis. Imaging was performed under a secondary electron mode using an operative voltage of 5 kV. The SEM instrumentation was equipped with an energy-dispersive X-ray spectroscopy (EDS) detector. The EDS detector was used to obtain information on the elemental composition of the fibers’ surface. The diameter of fibers was determined from the respective SEM images by ImageJ software (U.S. National Institutes of Health, Bethesda, MD, USA). The mean fiber diameter was calculated from the diameters of 80 individual fibers.

The thickness of the electrospun mats was determined using a micrometer with an accuracy of 0.01 mm. Five measurements were made and averaged for each mat. The standard deviations from the averaged values were also calculated.

The surface roughness was determined for the PHBV films modified under the same conditions as the PHBV electrospun mats. An atomic force microscope (AFM, Topometrix Explorer, Santa Clara, CA, USA) in contact mode in the air was used for the image scanning of the film surfaces. The AFM instrument operated in a constant-force regime. Three measurements were taken for each film, and the results are presented with arithmetic means.

The thermal properties were determined using a DSC1 differential scanning calorimeter (DSC, Mettler Toledo, Greifensee, Switzerland). The instrument was calibrated using high-purity n-octane, indium, tin, and zinc. An empty aluminum pan was used as a reference. The specimens were heated under a nitrogen atmosphere from −65 to 190 °C at a heating rate of 20 °C/min. DSC data analysis was performed with the Mettler-Toledo STARe 15.00 software. The degree of crystallinity for the PHBV mats was calculated using the following equation:

$$\chi_{c}\left(\%\right)=\frac{\Delta H_{m}}{\Delta H_{m100}}\times 100$$

where *χ_c_* is the degree of crystallinity, Δ*H_m_* is the measured enthalpy change of melting, and Δ*H_m_*_100_ is the melting enthalpy of the 100% crystalline polymer (128.0 J/g) [56].

Fourier transform infrared (FTIR) spectra were recorded using a JASCO FT/IR-6700 (JASCO Co., Ltd., Tokyo, Japan) spectrometer, which was equipped with an ATR attachment containing a single-reflection diamond crystal (ATR PRO670H-S). The ATR accessory was used with an angle of incidence of 45°. The penetration depth at 1000 cm^–1^ was approximately 1.66 μm. The spectra were collected over a range of 400–4000 cm^–1^ with a resolution of 4 cm^–1^ and 64 scans for signal accumulation.

The static sessile drop method was used to measure the contact angle of a pure water drop on the surface of unmodified and modified PHBV films. The measurements were performed at room temperature by using an automatic contact angle meter (CAM 101, KSV Instruments Ltd., Helsinki, Finland). For each sample, five measurements were performed at different spots of the film surface. The contact angle values presented here are the average of these five measurements.

### 2.5. In Vitro Assays and Microscopic Observation of Cell Morphology

The in vitro cytotoxicity of the PHBV mats was evaluated according to ISO 10993-5:2009. The procedure regarding the direct contact test was followed using a cell line of L929 fibroblast-like cells.

An MTS assay was used to assess cell viability/proliferation on the untreated and LiBH_4_-treated surfaces. For this purpose, the human SaOS-2 osteosarcoma cell line (HTB-85, ATCC, Gaithersburg, MD, USA) was used. The electrospun fiber mats were cut into circular pieces of 0.8 cm diameter and placed into the wells of a 48-well tissue-culture plate (clear flat bottom, Falcon, Corning, NY, USA). The samples were sterilized by exposure to UV irradiation in a laminar flow chamber for 10 min. The SaOS-2 cells were seeded onto the electrospun mats at 10,000 cells per well and incubated at 37 °C in a 5% CO_2_ atmosphere. The cells were grown in α-MEM medium (Cat. No. 22561054, ThermoFisher Scientific, Waltham, MA, USA) supplemented with 10% fetal bovine serum (FBS, Cat. No. 10270106, ThermoFisher Scientific, Waltham, MA, USA) and 1% penicillin-streptomycin antibiotic solution (PenStrep, Cat. No. 15070063, ThermoFisher Scientific, Waltham, MA, USA). After 24 h, the culture medium was replaced with fresh growth medium. The culture was then continued at 37 °C in 5% CO_2_ for 7 days. As a control group, the cells were seeded directly on the bottom of the wells. All cultures were carried out in triplicate. After a 7-day culture period, 100 μL of MTS reagent was added to each well, and then the plate was incubated for 45 min at 37 °C. The absorbance of the formazan dye produced was measured at 492 nm using a SpectraMax iD3 microplate reader (Molecular Devices, San Jose, CA, USA). For SEM imaging, the wells were washed thrice with PBS, and the cultures were fixed with 4% formaldehyde. After another triple wash with PBS, the cell-seeded scaffolds were dehydrated using increasing concentrations of ethanol and transferred to a critical point dryer (CPD, E3100, Quorum Technologies, Laughton, UK). The dry specimens were sputter-coated with gold. Cell imaging was performed using a JEOL JSM-5410 scanning electron microscope at 15 keV in the secondary electron-imaging mode.

### 2.6. Statistical Analysis

The data obtained from SEM micrographs, surface roughness measurements, and the MTS assay were statistically analyzed by one-way ANOVA followed by Tukey’s HSD test to determine whether a significant difference existed between the samples. The results were considered to be statistically significant at *p* ≤ 0.05.

## 3. Results

**Figure 1:**
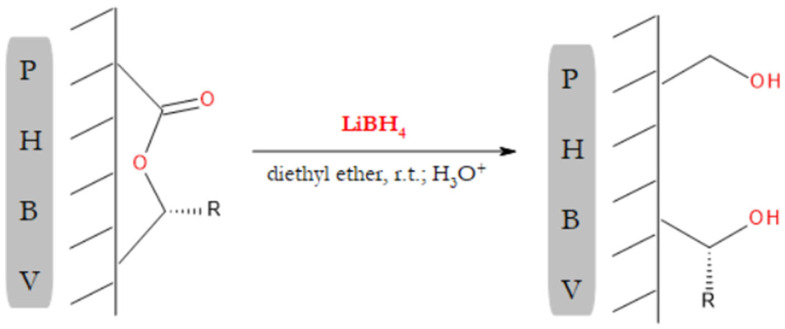
Scheme illustrating the modification of PHBV surface using LiBH_4_. The surface hydroxyl groups were generated through the reduction of the backbone ester groups: R = CH_3_ or C_2_H_5_.

The aim of our study was to introduce hydroxyl groups on the surface of PHBV electrospun fibers, which was supposed to decrease their hydrophobicity. The hydrophobicity nature of PHA’s surface is a well-known issue that restricts the use of PHAs in tissue-engineering applications. The surface enrichment with hydroxyl groups was to be accomplished by means of LiBH_4_ reduction of the PHBV ester bonds. The modification was conducted in a heterogeneous system, in which the PHBV mat constituted the solid phase, while the liquid phase was an ether solution of LiBH_4_. A schematic illustrating the introduction of surface hydroxyl groups is shown in Figure 1.

### 3.1. Average Molar Mass of the PHBV Mats before and after the LiBH_4_ Treatment

**Figure 2:**
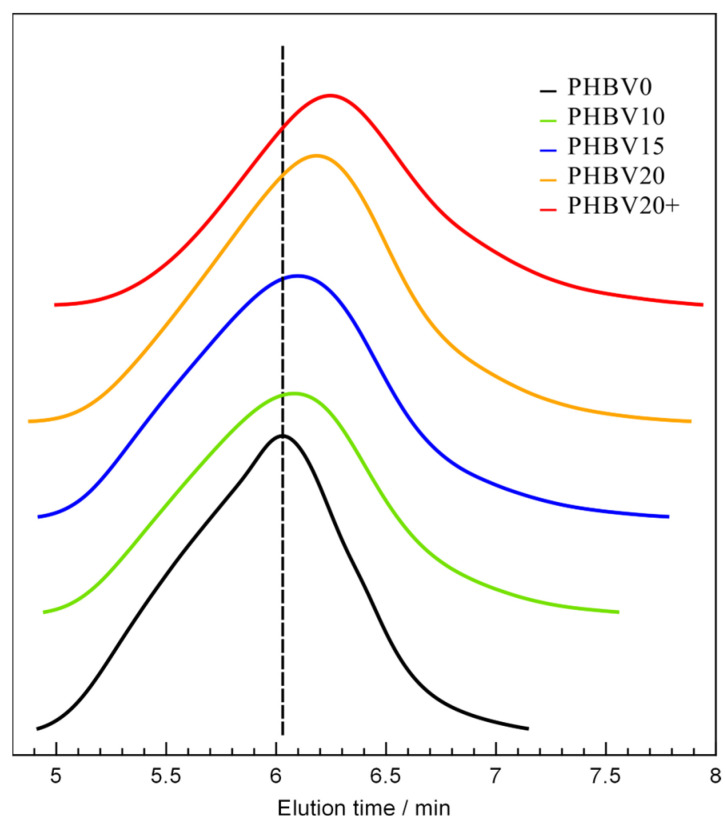
GPC chromatograms for the untreated and LiBH_4_-treated PHBV electrospun fibers.

As the lithium borohydride reduction of PHBV is a degradation reaction, the average molar mass of the mat’s material should change due to the LiBH_4_ treatment. Therefore, the blank sample and the modified mats were subjected to GPC analysis. The results of this analysis are shown in Table 2. The corresponding GPC chromatograms are shown in Figure 2.

The GPC results indicated that the average molar masses (M_n_ and M_w_) of the fibrous mat made of PHBV decreased with the duration of borohydride reduction. A decrease in the molar mass was also observed as a result of increasing the concentration of the LiBH_4_ solution. These results agreed with those obtained by us from the study on the LiBH_4_ reduction of PHB in the form of a powder [53]. However, in the case of PHB powder, a decrease in dispersity of the polymer was also observed [53]. The degradation of the PHBV mats was, in turn, accompanied by an increase in the material’s molar mass distribution. However, the reduction of PHB was carried out to obtain its low molar mass oligomers. In other words, the borohydride reduction of PHB was intended to degrade the whole mass of the powder. In the case of the PHBV fibers, only the surface was expected to be modified. Therefore, the fiber modification by LiBH_4_ was carried out under much milder conditions than used in the reduction of the powder. As a result of random chain scission, hydroxyl-terminated PHBV oligomers were formed on the mat surface. Thanks to the mild conditions, the average molar masses of these oligomers were still high. Therefore, the formed macrodiols remained on the fiber surface, which translated into the observed increase in the dispersity.

The decrease in weight-average molar mass was rather small when the reduction was carried out for up to 20 min and at a concentration of the borohydride solution equal to 0.005 mol/dm^3^. If the degradation was conducted for 20 min in a 0.0075 M solution of LiBH_4_, the decrease in the molar mass was more pronounced. Such a substantial decrease may indicate that the degradation of PHBV chains occurs in deeper and deeper layers of the fibers, which is undesirable. Therefore, a reaction time of 20 min at a LiBH_4_ concentration of 0.005 mol/dm^3^ was determined as the upper limit of the fiber modification conditions. It is also worth mentioning that the modification with LiBH_4_ practically did not cause a change in the mass of the fibers (the mass loss was less than 0.4%). In other words, no erosion was observed in the fibers due to the borohydride reduction.

### 3.2. Effect of LiBH_4_ Treatment on the Morphological Properties of Modified Materials

**Figure 3:**
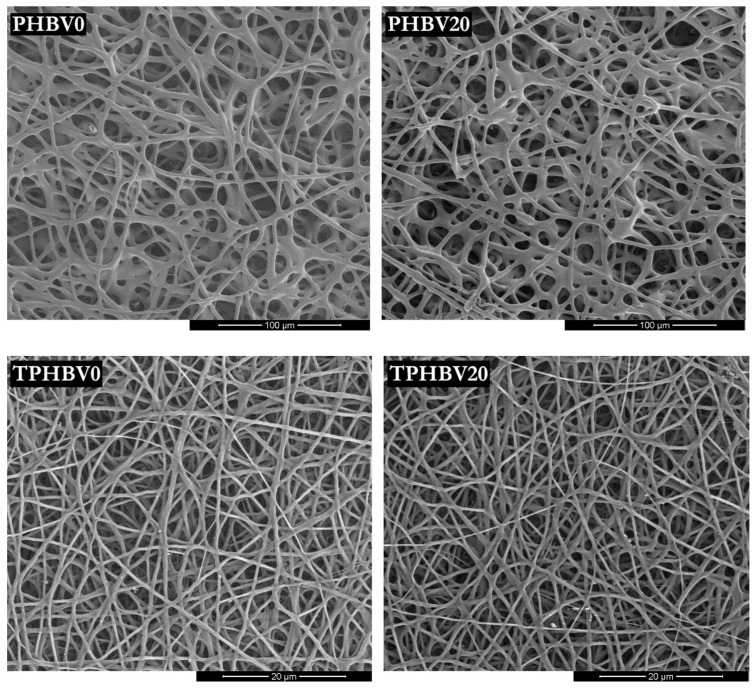
SEM images of the untreated PHBV fibers (TPHBV0 and PHBV0) and the treated ones. The treated fibers shown here correspond to the modification carried out for 20 min at a LiBH_4_ concentration of 0.005 mol/dm^3^. The modified fibers with a mean diameter of 0.74 ± 0.20 µm are denoted as TPHBV20, while the treated fibers of a larger diameter (3.75 ± 0.67 µm) are denoted as PHBV20.

The results of the molar mass measurements indicated that the degradation of PHBV microfibers could be limited to its outer layers. However, this required performing the modification under a low concentration of the reducing agent and within short time periods. This was important since the degradation affecting the inner layers of fibers may worsen their mechanical properties and eventually cause the destruction of the fibrous structure [31]. The fiber diameter and surface roughness measurements also supported the benign nature of the modification procedure. Both parameters were determined for the blank samples and the LiBH_4_-modified ones. The root-mean-square (RMS) surface roughness values were calculated not for the fibers but the PHBV film prepared by the solvent-casting method. Unlike casted films, fibrous mats have a highly porous structure, which makes it very difficult to measure their surface roughness. The determined RMS surface roughness and fiber diameters before and after the borohydride modification are given in Table 3. SEM images of the unmodified and modified electrospun PHBV fiber mats can be seen in Figure 3.

As seen from the SEM images, the LiBH_4_ reduction of PHBV conducted for up to 20 min at a concentration of 0.005 mol/dm^3^ did not destroy the fibrous structure. Even the mat composed of fibers with a mean diameter of less than 1 µm (0.74 ± 0.20 µm; TPHBV) retained its fibrous structure after the 20-minute treatment. Furthermore, the reaction with LiBH_4_ virtually did not induce a change in the mean fiber diameter of the mat made up of much larger fibers. The fiber diameter measurements indicated that the fibers with an arithmetic mean diameter of 3.75 ± 0.67 µm were subjected to LiBH_4_ reduction. As a result of the modification, PHBV fibers with diameters ranging from 3.79 ± 0.83 µm to 4,06 ± 0.78 µm were obtained. However, any differences in fiber diameter between the blank sample and the modified mats were statistically insignificant. The mean fiber diameter of the TPHBV mat also remained unchanged after treatment with LiBH_4_. The diameter of the fibers does not always remain unchanged after chemical degradation. A decrease in fiber diameter was observed in the case of polyethylene terephthalate (PET) nonwoven that was hydrolyzed in an aqueous solution of NaOH [57]. This hydrolysis was further accompanied by a double-digit weight loss of the modified fibers. The surface modification described here resulted in fibers with both the diameter and the weight virtually unchanged. It testifies the mildness of the adopted modification conditions, which is important for preserving the mechanical properties of the fibers.

**Figure 4:**
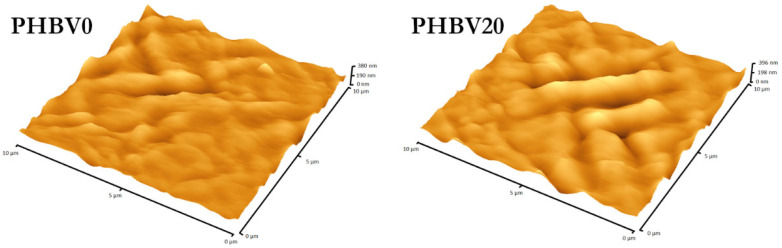
AFM images of the untreated film surface (PHBV0) and the film treated with a 0.005 M LiBH_4_ solution for 20 min (PHBV20).

One of the basic quantities characterizing the morphology of a surface is the RMS roughness (also denoted R_q_). It can be defined as the root-mean-square (RMS) deviation of the position of surface points with respect to the mean surface level. As already mentioned, the RMS parameter was determined for the surface of the PHBV films. The results of these determinations are given in Table 3. They indicated that the film’s surface roughness remained virtually unchanged due to the reaction with LiBH_4_ (the differences in the RMS roughness values were statistically insignificant). A slight increase in surface roughness was observed only in the case of the film treated for 20 min (PHBV20). These results supported the conclusions drawn from the GPC data, namely the lack of erosion observed under the applied conditions. Such changes in the surface roughness are important for yet another reason. Several studies have found that surface roughness may affect cell adhesion behavior [30,58]. Since the surface roughness remained almost unchanged, a change in the number of adherent cells on the LiBH_4_-treated surfaces can be mainly attributed to the change in the scaffold’s surface chemistry. In contrast to the method described here, the approaches that involve hydrolysis or aminolysis of polyester chains usually result in a significantly higher increase in surface roughness [59,60]. It has been reported that alkaline-hydrolyzed and aminolyzed polylactic acid (PLA) films showed at least 13 times higher surface roughness than untreated film [60]. The AFM images of the film surface before and after the 20-minute reaction with LiBH_4_ are shown in Figure 4.

### 3.3. Thermal Properties of LiBH_4_-Modified PHBV Fibrous Mats

Differential scanning calorimetry (DSC) was utilized to examine the effect of LiBH_4_ reduction on the thermal properties of the nonwoven material. A typical DSC measurement involves two pre-programmed heating cycles. The first heating cycle refers to the thermal history of the sample under examination. The shape of the first heating curve for the PHBV mats may be affected by the processes such as electrospinning, LiBH_4_ degradation, and drying at 37 °C. Therefore, this curve should be different for the mats modified after various reaction times (these curves as well as the second heating thermograms can be found in the Supplementary Information, Figures S1 and S2). The first heating DSC thermograms were used to determine the crystallinity degree and melting temperatures of the modified PHBV mats. The second ones were used in turn to define the glass transition temperature. The determined thermal properties for the unmodified and modified mats are given in Table 4 (thermal properties obtained from the second DSC heating scans are included in the Supplementary Information, Table S1).

It can be seen from Table 4 that the degree of crystallinity in the mats increased with the modification time. This was not surprising since the average molar mass of the mat material also decreased with increasing the reduction time, as revealed by the GPC (see Table 2). A gradual increase in crystallinity would not occur if the reaction with LiBH_4_ proceeded only within a crystalline phase. The degradation of the amorphous phase resulted in a reduction in the length of their chains. According to the literature, shorter chains are more likely to crystallize than those with a longer length [61]. This may lead to the ordering of previously unordered polymer regions, that is, increasing the degree of crystallinity in a polymer. In other words, new crystalline entities were generated from the degraded amorphous chains. Since the crystallization rate increased with increasing temperature, the ordering of the PHBV structure could have occurred during the 40-hour drying of the mats at 37 °C. The drying process was carried out to remove the residual solvents.

By comparing the data in Table 4, it can be concluded that the glass transition and melting temperature of PHBV decreased slightly with increasing the reduction time. As determined by DSC, the T_g_ and two T_m_ values before the modification were 5.2, 141.5, and 159.3 °C, respectively. After the 20-minute LiBH_4_ reduction, the scaffold material exhibited a value of T_g_ of 3.7 °C and T_m_ of 139.5 and 159.1 °C. The changes in the transition temperatures of PHBV were most likely due to the decrease in its average molar mass. Obviously, the drop in the molar mass resulted from the reaction of PHBV with LiBH_4_. The small value of the mentioned changes suggested that the fiber degradation was confined to the outer layers. Unlike the T_g_ and T_m_ of PHBV, its degree of crystallinity became higher after the LiBH_4_ treatment. The increase in crystallinity was probably due to the decrease in the average molar mass of PHBV. Such a relationship between crystallinity and average molar mass was reported by several studies [61]. The studies described in the literature indicated that crystallinity affects the extent of cell adhesion [62]. The data concerning the effect of LiBH_4_ reduction on the crystallinity of the scaffold would be helpful for its potential use in future scaffold design.

The above-mentioned changes in the thermal properties of the mat material are also indicative of the effective removal of lithium chloride, which is a byproduct of the reaction of PHBV with LiBH_4_. Lithium chloride was found on the mat’s surface before washing it with ethanol. The identification was carried out by SEM equipped with an energy-dispersive X-ray spectroscopy (EDS) detector (the respective EDS spectra are presented in the Supplementary Information, Figure S3). Experimental studies reported in the literature have shown that even the low amounts of lithium chloride (0.5 wt%) present in the polymer sample exhibit plasticizing properties [63,64]. The presence of lithium chloride is manifested by lowering the melting point and the crystallinity of a polymer. However, the crystallinity of PHBV was higher after the treatment with LiBH_4_. The washing out of lithium chloride was essential due to its high hygroscopicity. Water absorbed by this compound could falsify the wetting angle measurement of the modified material.

### 3.4. Infrared (IR) Spectra of Surfaces Treated with LiBH_4_ Ethereal Solution

**Figure 5:**
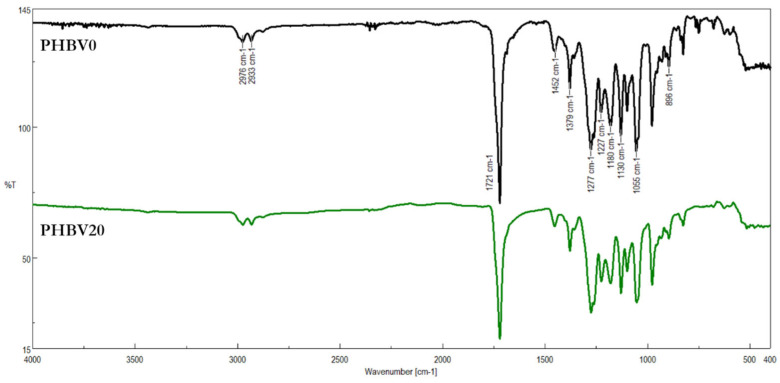
ATR-FTIR spectra of the unmodified (PHBV0) and the modified electrospun fibers (PHBV20).

Fourier transform infrared spectroscopy (FTIR) is a technique commonly used to identify the functional groups present in a sample under investigation. The method that allows obtaining the IR spectrum from the near-surface region is known as attenuated total reflection (ATR). Its unquestionable advantage is that no sample pretreatment is required. The surfaces of the electrospun mats before and after the modification were directly subjected to ATR-FTIR. Figure 5 shows the infrared absorption spectrum of the untreated mat and the mat treated with LiBH_4_ for 20 min.

In the IR spectrum of the blank sample (PHBV0), the absorption bands characteristic for polymers of hydroxyalkanoic acids could be found. For example, the band located at 1721 cm^–1^ was due to the C=O stretching vibration of PHBV; the bands at 2976 cm^–1^ and 2933 cm^–1^ belonged to the symmetric stretching vibration of –CH_3_ and asymmetric stretching vibration of –CH_2_, respectively. The vibrational bands of the C–O–C bond in PHBV appeared at 1277, 1180, and 1130 cm^–1^, among others [65,66]. As can be seen in Figure 5, the oscillatory-rotational spectrum of the untreated nonwoven material was virtually the same as the spectrum of the LiBH_4_-modified fibers. The degradation of PHBV due to reaction with LiBH_4_ should produce macromolecules containing primary and secondary hydroxyl end groups. In our previous works, the formation of such macromolecules was confirmed by ^1^H NMR spectroscopy and ESI-MS spectrometry [53,67]. However, the bands corresponding to hydroxyl groups were barely noticeable in the IR spectrum of the modified mats. This was consistent with the findings of Chen and McCarthy, who modified the surface of polyethylene terephthalate (PET) film with LiAlH_4_ [68]. They also reported no occurrence of the mentioned peaks in the IR spectra. They explained that the absence of IR bands for OH groups was due to an insufficient depth at which the reduction with LiAlH_4_ takes place. Probably for the same reason, the IR spectra of the LiBH_4_-modified fibers had no O–H absorption bands. As mentioned in the experimental section, the penetration depth into the sample was 1.66 μm. The fibers that were subjected to the borohydride degradation had an average diameter of 3.75 ± 0.67 μm. If the degradation only affected the outer layers of the fibers, the relative amount of OH groups could not be large enough to be detected by the ATR-FTIR technique. Therefore, the difficulties in the identification of the O–H vibrational bands may indicate that the modifications were confined to the outer layers of PHBV fibers.

### 3.5. Wettability of the Modified PHBV Films

The main purpose of a scaffold is to facilitate cell attachment onto its surface [30,69]. It largely depends on the surface properties of materials, such as surface energy, roughness, morphology, chemical composition, and wettability [28,70,71]. Among them, the latter is of particular interest as numerous experimental studies have demonstrated the correlation between hydrophilicity and the cell adhesion efficiency [37,70,72,73]. The highest cell adhesion occurred on moderately hydrophilic surfaces [37,72]. Importantly, this rule does not depend on the type of cultured cells [72]. A static contact angle was employed as the measure of surface wettability. The contact angle measurement of the surface of a porous mat is, for technical reasons, much more difficult than for a non-porous film. Therefore, the measurements were carried out for the LiBH_4_-modified PHBV films instead of the electrospun mats. The obtained results are summarized in Table 5.

**Figure 6:**
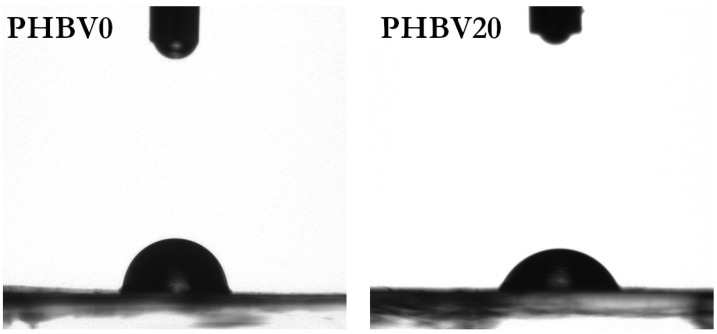
Optical contact angle images of water on the untreated film surface (PHBV0) and the LiBH_4_-treated one (PHBV20).

As evident from Table 5, the hydrophilicity of the PHBV films increased with increasing the degradation time and the initial concentration of LiBH_4_. The surface of the unmodified film showed a contact angle of 86.6 ± 2.3°. The contact angle on the surface of neat PHBV decreased to 76.7 ± 1.7°, 73.1 ± 1.4°, 72.3 ± 1.3°, and 64.5 ± 1.9° after the degradation lasted for 5, 10, 15, and 20 min, respectively. These values corresponded to an initial LiBH_4_ concentration of 0.005 mol/dm^3^. When the concentration was increased to 0.0075 mol/dm^3^, the contact angle decreased from 72.3 ± 1.3 to 67.7 ± 1.8°. Thus, the hydrophobic PHBV surface became hydrophilic due to the borohydride treatment. Furthermore, the results showed that the wettability of LiBH_4_-degraded surfaces could be tailored by simply changing the degradation conditions. This made it possible to optimize the surface properties for cell culture purposes. As mentioned earlier, the borohydride reduction of PHBV film resulted in almost no increase in the film’s surface roughness (a slight increase was observed only for the PHBV20 film). This was revealed by AFM analysis. As is known, surface roughness affects the wetting of a solid surface. Therefore, the decrease in the water contact angles of PHBV films can mainly be attributed to changes in the surface chemistry and not to changes in the surface roughness. In other words, it was evidence that the LiBH_4_ treatment of PHBV film resulted in the occurrence of additional surface hydroxyl groups. The change in surface chemistry was the primary goal of the modification method presented here. Figure 6 shows the images of a water drop placed on the unmodified and modified surface of the PHBV film.

### 3.6. Cytotoxicity Evaluation

**Figure 7:**
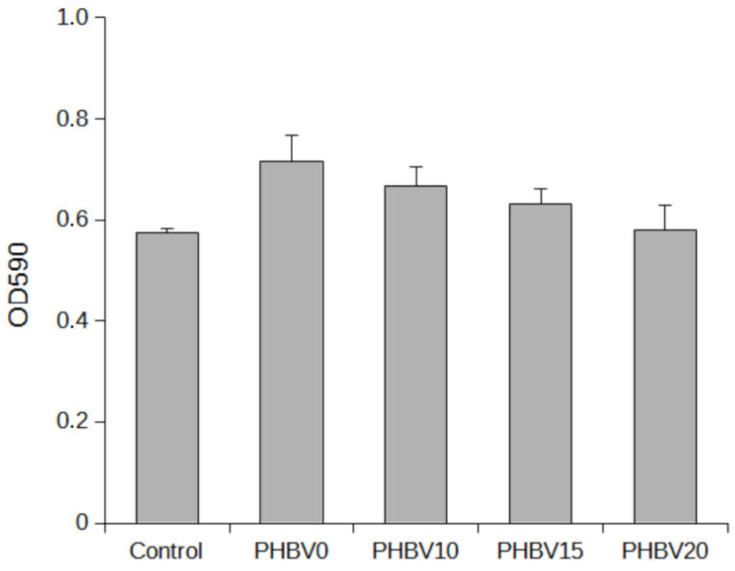
Graph representing the results obtained with L929 fibroblast cells in the evaluation of cytotoxicity of the modified PHBV fibrous mats.

Before being subjected to the MTS proliferation assay, the untreated and LiBH_4_-treated PHBV electrospun fibers were evaluated for their cytotoxic effects. The in vitro cytotoxicity test was performed according to the ISO standards (10993-5, 2009). These standards define cytotoxic materials as those that reduce cell viability by more than 30% (the control sample is considered 100% viability). The cytotoxicities were evaluated in a L929 fibroblast cell line, and the results are shown as a graph in Figure 7. These results showed that the PHBV mats modified through the reaction with LiBH_4_ were not cytotoxic.

### 3.7. MTS Cell Proliferation Assay

The aim of this study was to employ borohydride reduction to introduce hydroxyl groups onto the surface of PHBV fibers. As a result, the hydrophilicity of the fibers was supposed to be improved. According to scientific reports, the introduction of surface hydroxyl groups into hydrophobic materials significantly enhances their cell adhesive properties [44]. It should be mentioned here that cells rarely adhere directly to the surface of scaffold material. In in vitro cultures, cell adhesion is preceded by the adsorption of proteins from the culture medium. The cells then adhere to such a protein layer adsorbed on the scaffold surface [37,74,75]. However, cell adhesion does not depend on the quality and quantity of adsorbed proteins but rather on the conformation they adopt on the adsorption to a solid [25,37,75,76,77,78]. Hence, the scaffold surfaces are required to be moderately hydrophilic. Surfaces that are too hydrophilic do not induce proteins to adopt the conformation best suited for subsequent cell adhesion [25,78,79].

**Figure 8:**
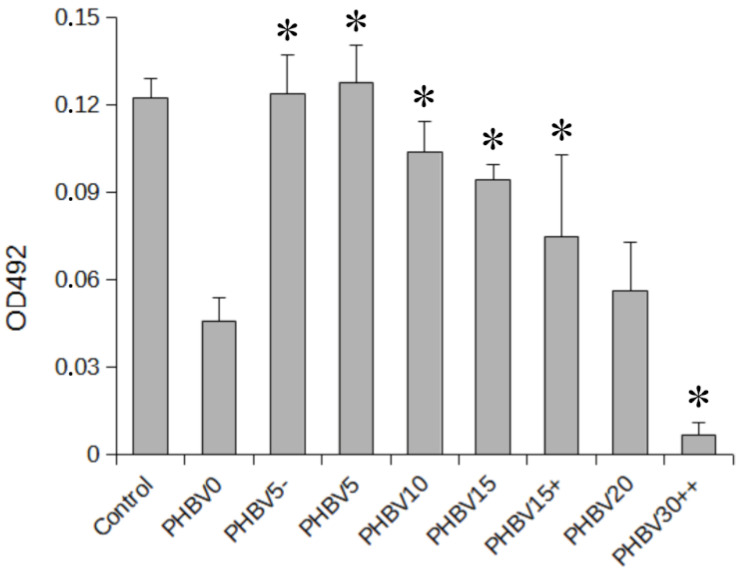
MTS results of SaOS-2 cells on the unmodified and LiBH_4_-modified PHBV fibers after 7 days of cell seeding. An asterisk (*) indicates significant differences (*p* ≤ 0.05) in optical density between the modified and non-modified samples.

As revealed by contact angle measurements, the hydrophobic surface of the PHBV film became hydrophilic after the LiBH_4_ treatment. Therefore, the modified fibers should have elicited a better cellular response than the unmodified ones. The effect of the reaction time and LiBH_4_ concentration on the cellular response of the PHBV mats was determined using an MTS assay. The assay was carried out by the use of a human osteoblast-like cell line (SaOS-2). The cells were first seeded onto the unmodified and functionalized PHBV mats and then cultured in a serum-containing medium (SCM) for 7 days. The results obtained by using the MTS assay are shown in the form of a column graph in Figure 8. The graph shows the metabolic activity of SaOS-2 cells in response to 7-day exposure to the PHBV mats.

The results obtained from the MTS assay showed that all the PHBV fibrous mats treated for up to 15 min with a LiBH_4_ solution at a concentration not higher than 0.0075 mol/dm^3^ had a better ability to support osteoblast viability and proliferation than the untreated mat. The highest number of SaOS-2 cells was found to be attached to the surface that was modified within 5 min in either 0.005 M (PHBV5) or 0.0025 M (PHBV5−) LiBH_4_. There was no difference in the metabolic activity of SaOS-2 cells cultured for 7 days on the mentioned surfaces. It is worth noting that the viability of those cells was comparable to the control group of SaOS-2 population grown directly on the bottom of a 48-well plate. This suggests the appropriate adhesive properties of the PHBV5 and PHBV5− mats to culture adherent cells on their surfaces. In contrast, the mats designated PHBV10, PHBV15, and PHBV20 showed a successively lower capacity to promote SaOS-2 cell proliferation compared to the PHBV5 and PHBV5− mats. In the case of the PHBV20 mat, the cell proliferation was comparable to that of the untreated mat. Increasing the concentration of the LiBH_4_ solution also caused a reduction in the number of cells attached to the PHBV fibers. This was indicated by comparing the MTS result obtained for the PHBV15 mat with that obtained for the PHBV15+ sample. Therefore, the degradation of PHBV fibers carried out in a 0.005 M or 0.0025 M LiBH_4_ solution for 5 min was found to be the most beneficial for improving the biological properties of the fibers.

The observed deterioration in the adhesive properties of the fibers was most likely caused by the unfavorable spatial structure of the proteins adsorbed on their surface. As mentioned earlier, cell adhesion is usually preceded by protein adsorption on the scaffold surface. The non-optimal conformation of proteins can be, in turn, explained by excessive changes in the surface properties of the modified electrospun fibers. As can be seen in Figure 8, the cell proliferation results correlated well with the extent of PHBV degradation. On the other hand, the contact angle measurements showed that the higher the level of PHBV degradation, the higher the surface hydrophilicity. Therefore, one can say that the surfaces of the PHBV10, PHBV15, PHBV15+, and PHBV20 fibrous mats were too hydrophilic to induce the optimal protein conformation for subsequent cell adhesion and proliferation. Conversely, the surface wettability of the PHBV5 fabric was good enough to demonstrate the best cell-attachment performance. The influence of surface hydrophilicity on cell attachment was particularly evident for the mat treated for 30 min with a solution containing a high concentration of LiBH_4_ (0.02 M; PHBV30++). The PHBV30++ mat was so hydrophilic that almost no cells were observed to adhere to its surface after a 7-day culture period. This mat is an excellent example of how a very hydrophilic surface can negatively affect cellular attachment. It can be seen from the cell proliferation results that the cellular response could be controlled to some extent by changing the modification conditions.

**Figure 9:**
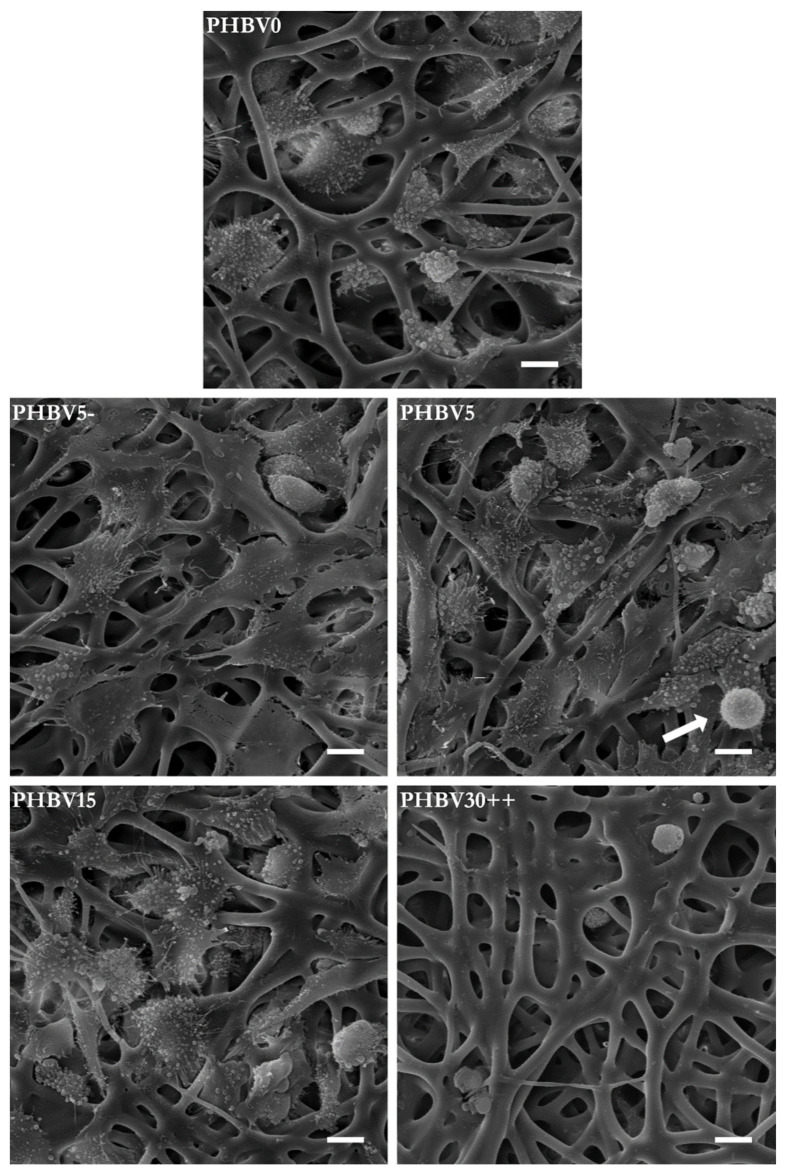
SEM images of SaOS-2 cells cultured for 7 days on the PHBV fibers modified under different reaction conditions (scale bars are all 10 μm). The white arrow on the image of the PHBV5 mat indicates the cell with mitotic morphology.

The results of the MTS assay were well consistent with the SEM observations. Figure 9 presents the selected SEM images of SaOS-2 cells cultured for 7 days on the PHBV fibrous mats (additional SEM images are provided in the Supplementary Information). The proliferative/metabolic activity measured with the MTS assay indicated that the number of cells attached to the material surface decreased with an increase in the concentration of LiBH_4_ and treatment time. The cells cultured on the PHBV5 mat occupied not only the scaffold pores but also the surface of the fibers. In contrast, the SEM images of the unmodified fabric (PHBV0) showed no cells attached to the fibers but were only present in the scaffold pores. The SEM images of the PHBV30++ sample revealed, in turn, that its surface was almost free of adhered cells after the culture period. What further distinguishes the cells that colonized fibers degraded under different conditions is the morphology. As seen in Figure 9, a typical cell morphology was noticed for the cells cultured on the mats that displayed the best adhesive properties (the PHBV5 and PHBV5− mats). Compared to this, the cells cultured on the PHBV15 and PHBV0 nonwoven material exhibited a more rounded morphology. Moreover, the filopodia and lamellipodia of these cells were less pronounced than those attached to the PHBV5 and PHBV5− mats. Based on these findings, one may conclude that surfaces with suitable properties for the attachment of SaOS-2 cells were those that induced marked cell flattening and spreading. This was not surprising, since osteoblasts need to adopt a flattened well-spread morphology to perform their biological functions, such as the production of ECM components. The SEM images also indicated the presence of numerous pseudopodia, thanks to which osteoblastic cells were able to attach to and migrate through the scaffold. Moreover, in the SEM image of the PHBV5 sample, one can see a cell undergoing mitotic division (it is marked with an arrow). The cell was spherical and surrounded by numerous filopodia. The presence of such cells is evidence that the surfaces of the LiBH_4_-modified fibers were an attractive site for cells to adhere to and proliferate.

## 4. Conclusions

The use of LiBH_4_ allowed for the modification of the surface chemistry of PHBV electrospun fibers. Thanks to the very mild conditions of the modification process, the LiBH_4_-modified mats retained their fibrous structure without any decrease in the mean fiber diameter. Moreover, when the PHBV films were subjected to the borohydride reduction, no change in the surface roughness was observed with increasing the reduction time and the LiBH_4_ concentration. Since the surface roughness remained unchanged, a change in the wettability of the film could mainly be attributed to the change in the surface chemistry. Thus, the observed increase in the hydrophilicity of the modified films was evidence of an increase in the density of polar surface groups. More importantly, the contact angle measurements indicated that the surface wettability could be controlled by changing the modification conditions. This allowed us to optimize the surface properties to achieve the highest cell proliferation ability. The MTS assay showed that the 5-minute treatment with a 0.005 M or 0.0025 M LiBH_4_ solution increased the number of attached cells on the mat surface by almost three times compared to the untreated fibers. In contrast, the fibers treated for 30 min with a more concentrated LiBH_4_ solution (0.02 M) were almost free of adhered cells. The SEM imaging revealed that the results of the MTS assay correlated with the observed cell morphology. When cultured on the mats that supported the best cell attachment (the PHBV5 and PHBV5− mats), the cells exhibited more flattened and spread morphologies than those cultured on the other mats.

The DSC studies revealed that the LiBH_4_-treated mats exhibited a higher degree of crystallinity than the unmodified mat. The crystallinity increased with the increase in the reaction time. This suggests that the modification preferentially occurred in the amorphous regions of the material. In conclusion, LiBH_4_ can be utilized as a reducing agent to prepare fibrous scaffolds with improved biological properties for tissue-engineering applications.

## Figures and Tables

**Table 1:** Denotation of the LiBH_4_-treated PHBV samples.

Sample	t [min]	c_LiBH4_ [mol/dm^3^]
TPHBV0 ^a^	0	0
TPHBV20	20	0.005
PHBV0 ^a^	0	0
PHBV5−	5	0.0025
PHBV5	5	0.005
PHBV10	10	0.005
PHBV15−	15	0.0025
PHBV15	15	0.005
PHBV15+	15	0.0075
PHBV20	20	0.005
PHBV20+	20	0.0075
PHBV30++	30	0.02

^a^ TPHBV0 and PHBV0 are the denotations of the blank samples.

**Table 2:** Average molar masses (M_n_ and M_w_) and dispersity (M_w_/M_n_) of the PHBV mats subjected to the reaction with LiBH_4_ under different conditions.

Sample	M_n_ [g/mol]	M_w_ [g/mol]	M_w_/M_n_
PHBV0	106,700	236,500	2.2
PHBV10	70,600	194,500	2.8
PHBV15	60,000	191,000	3.2
PHBV20	49,000	158,600	3.2
PHBV20+	35,400	158,600	3.2

**Table 3:** Film surface roughness and mean fiber diameter of the electrospun mats before and after treatment with LiBH_4_.

Sample	Mean Fiber Diameter ^a^ [nm]	Film RMS Roughness ^b^ [nm]
TPHBV0	740 ± 200	n/a
TPHBV20	760 ± 200	n/a
PHBV0	3750 ± 670	33 ± 6.0
PHBV10	4010 ± 930	36 ± 1.7
PHBV15	4060 ± 780	35 ± 4.0
PHBV15+	3790 ± 830	35 ± 3.5
PHBV20	3850 ± 760	43.3 ± 4.0 *

^a^ Mean value of eighty measurements ± standard deviation. Based on ANOVA results, there were no significant differences (*p* ≤ 0.05) between the samples; ^b^ Mean value of three measurements ± standard deviation. An asterisk (*) indicates significant differences (*p* ≤ 0.05) in surface roughness between the modified and non-modified films.

**Table 4:** Thermal properties and crystallinity of the electrospun PHBV mat after various times of degradation with LiBH_4_.

Sample	1st Heating ^a^				2nd Heating ^a^
	T_m1_ [°C]	T_m2_ [°C]	ΔH_m_ [J/g]	χ_c_ ^b^ [%]	T_g_ [°C]
PHBV0	141.5	159.3	98.8	77.2	5.2
PHBV10	139.1	158.2	100.2	78.3	4.6
PHBV15	136.2	157.9	103.4	80.8	4.0
PHBV20	139.5	159.1	105.2	82.2	3.7

^a^ The heating rate was 20 °C/min in the temperature range from −65 to 190 °C; ^b^ ΔH_m100_ = 128.0 J/g.

**Table 5:** Water contact angles of PHBV film modified with LiBH_4_ under different conditions.

Sample	Contact Angle ^a^ [°]
PHBV0	86.6 ± 2.3
PHBV5	76.7 ± 1.7
PHBV10	73.1 ± 1.4
PHBV15−	76.4 ± 1.3
PHBV15	72.3 ± 1.3
PHBV15+	67.7 ± 1.8
PHBV20	64.5 ± 1.9

^a^ Mean value of five measurements ± standard deviation.

## Data Availability

The raw data supporting the conclusions of this article will be made available upon request.

## Supplementary Materials

The following supporting information can be downloaded at: https://www.mdpi.com/article/10.3390/ma15217494/s1, Figure S1: DSC first heating thermograms of the untreated and LiBH_4_-treated PHBV fibrous mats; Figure S2: DSC second heating thermograms of the untreated and LiBH_4_-treated PHBV fibrous mats; Table S1: Thermal properties of the untreated and LiBH_4_-treated PHBV polymer as determined from the second DSC heating thermograms; Figure S3: EDS spectra and their corresponding element content. (A) The PHBV15 mat before washing it with ethanol. (B) The PHBV15 mat after ethanol washing; Figure S4. SEM images of SaOS-2 cells cultured for 7 days on the different modified PHBV fibers (scale bars are all 10 μm).
