# Supplementary material for: Surface Modification of PHBV Fibrous Scaffold via Lithium Borohydride Reduction
Source: Materials (Basel). 2022 Oct 25;15(21):7494. doi: 10.3390/ma15217494 (PMC9653721; doi:10.3390/ma15217494)
Supplement: Supplementary file 1 [file materials-15-07494-s001.zip › materials-1886790-supplementary.pdf]

Supporting Information

Surface modification of PHBV fibrous scaffold via lithium borohydride reduction

Paweł Chaber [1,*], Grzegorz Tylko [2], Jakub Włodarczyk [1], Paweł Nitschke [1], Anna Hercog [1], Sebastian Jurczyk [3], Jakub Rech [4], Jerzy Kubacki [5], Grażyna Adamus [1,*]

[1] Centre of Polymer and Carbon Materials, Polish Academy of Sciences; M. Curie-Skłodowska 34, 41-819 Zabrze, Poland; [2] Department of Cell Biology and Imaging, Institute of Zoology and Biomedical Research, Faculty of Biology, Jagiellonian University; Gronostajowa 9, 30-387 Kraków, Poland; [3] Institute for Engineering of Polymer Materials and Dyes, Łukasiewicz Research Network, Marii Skłodowskiej-Curie 55, 87-100 Toruń, Poland; [4] Department of Biotechnology and Genetic Engineering, Faculty of Pharmaceutical Sciences in Sosnowiec, Medical University of Silesia, Poniatowskiego 15, 40-055 Katowice, Poland; [5] University of Silesia in Katowice, Faculty of Science and Technology, Institute of Physics, 75 Pułku Piechoty 1, 41-500 Chorzów, Poland

Corresponding authors: pchaber@cmpw-pan.pl; gadamus@cmpw-pan.pl

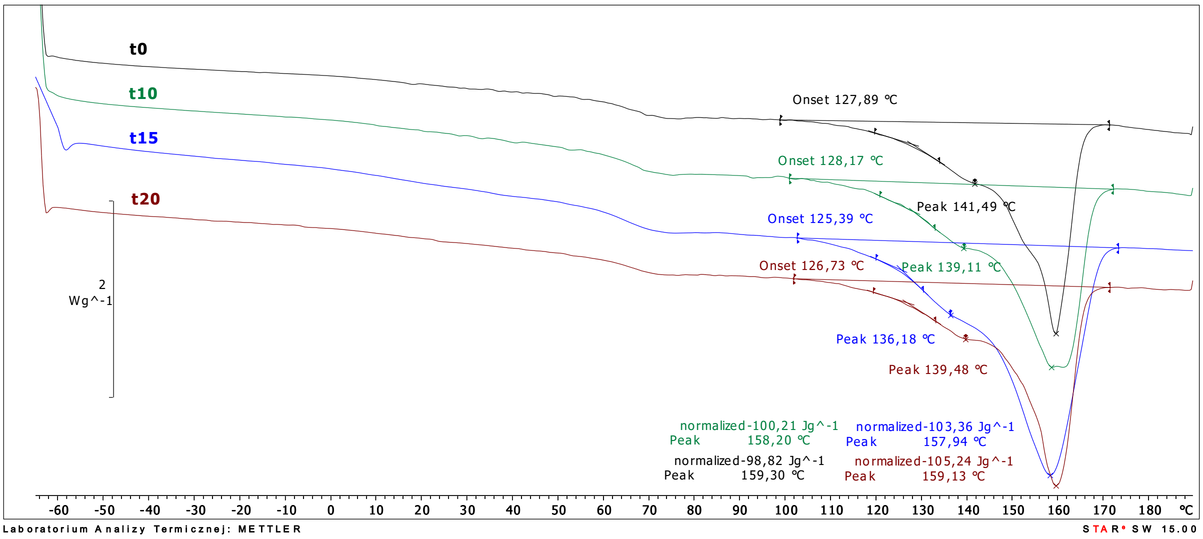

Figure S1. DSC first heating thermograms of the untreated and $LiBH_4$-treated PHBV fibrous mats.

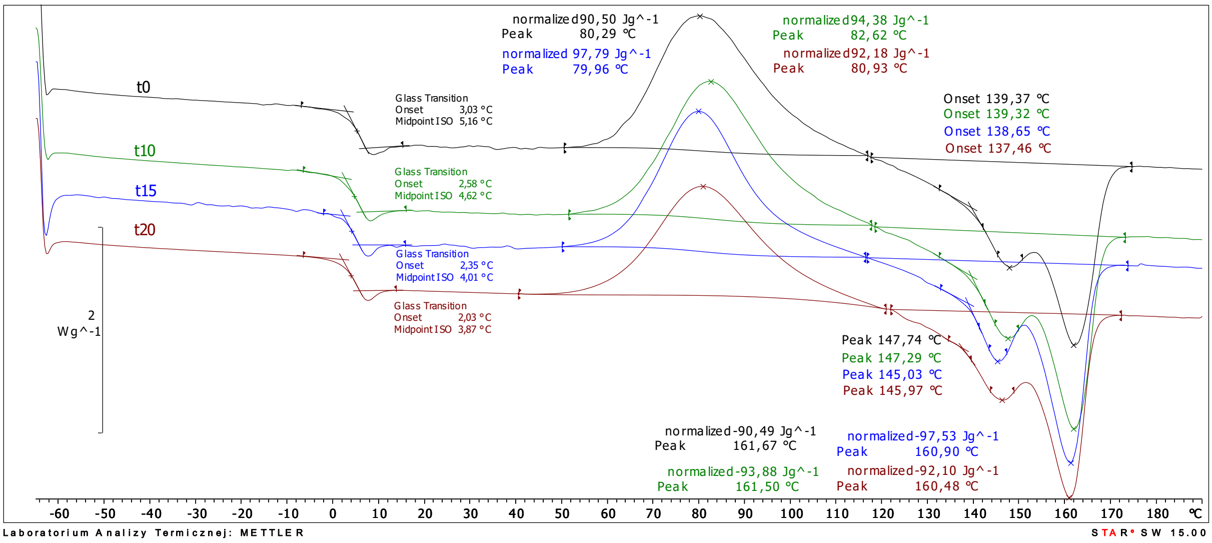

Figure S2. DSC second heating thermograms of the untreated and $LiBH_4$-treated PHBV fibrous mats.

Table S1. Thermal properties of the untreated and $LiBH_4$-treated PHBV polymer as determined from the second DSC heating thermograms

**Sample**	**2nd Heating**[a]				
	T_g **[°C]**	T_{m1} **[°C]**	T_{m2} **[°C]**	ΔH_m **[J/g]**	χ_c[b] *[%]*
PHBV0	5.2	147.7	161.7	89.8	70.2
PHBV10	4.6	147.3	161.5	93.3	72.9
PHBV15	4.0	145.0	160.9	97.2	75.9
PHBV20	3.7	146.0	160.5	93.8	73.3

[a] The heating rate was 20°C/min in the temperature range from −65 to 190°C; [b] ΔH_{m100} = 128.0 J/g

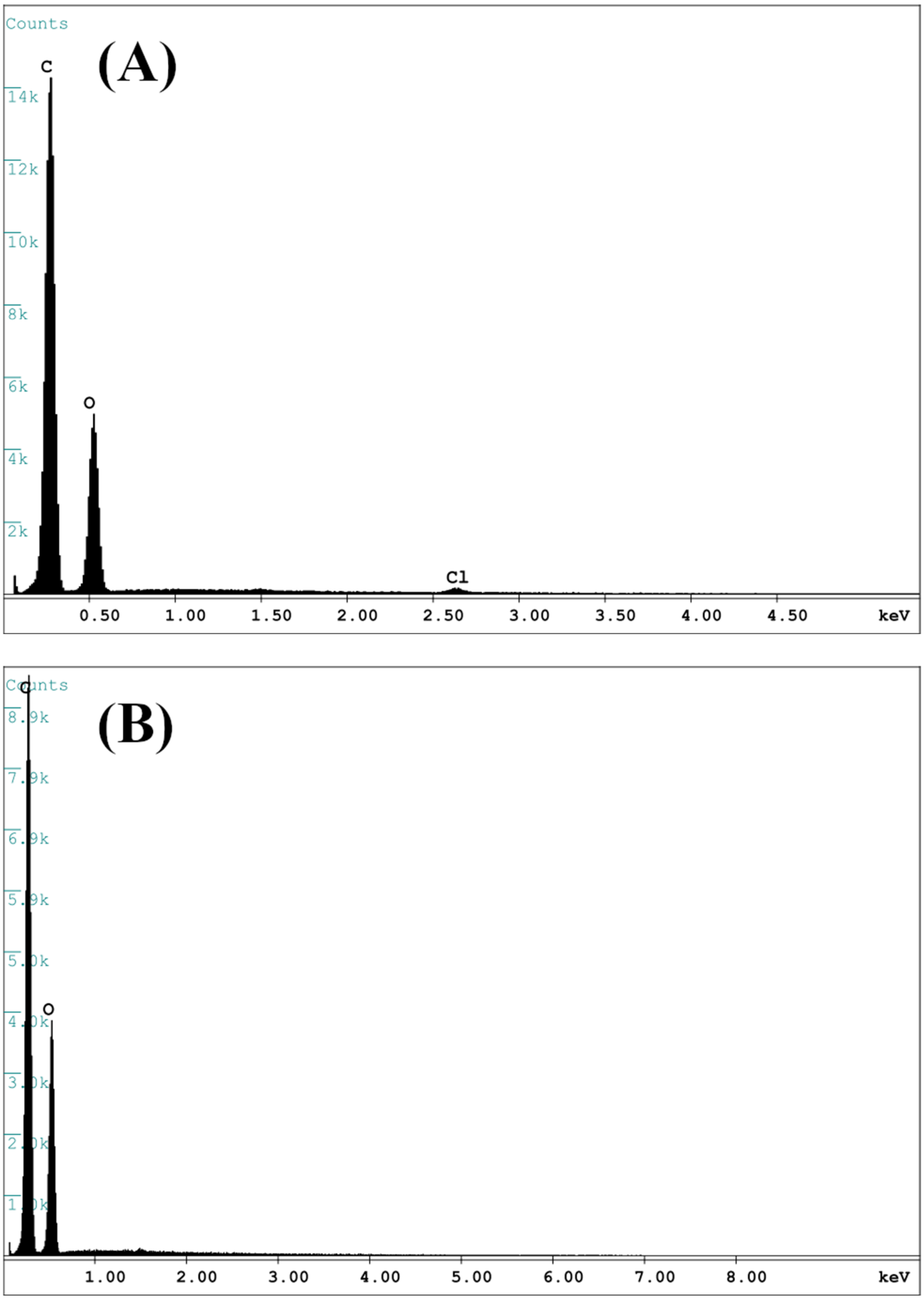

Figure S3. Representative EDS spectra obtained by X-ray microanalysis of (A) PHBV15 mat before washing it with ethanol and (B) PHBV15 mat after ethanol washing. Please note that ethanol washing effectively removes LiCl from the material surface.

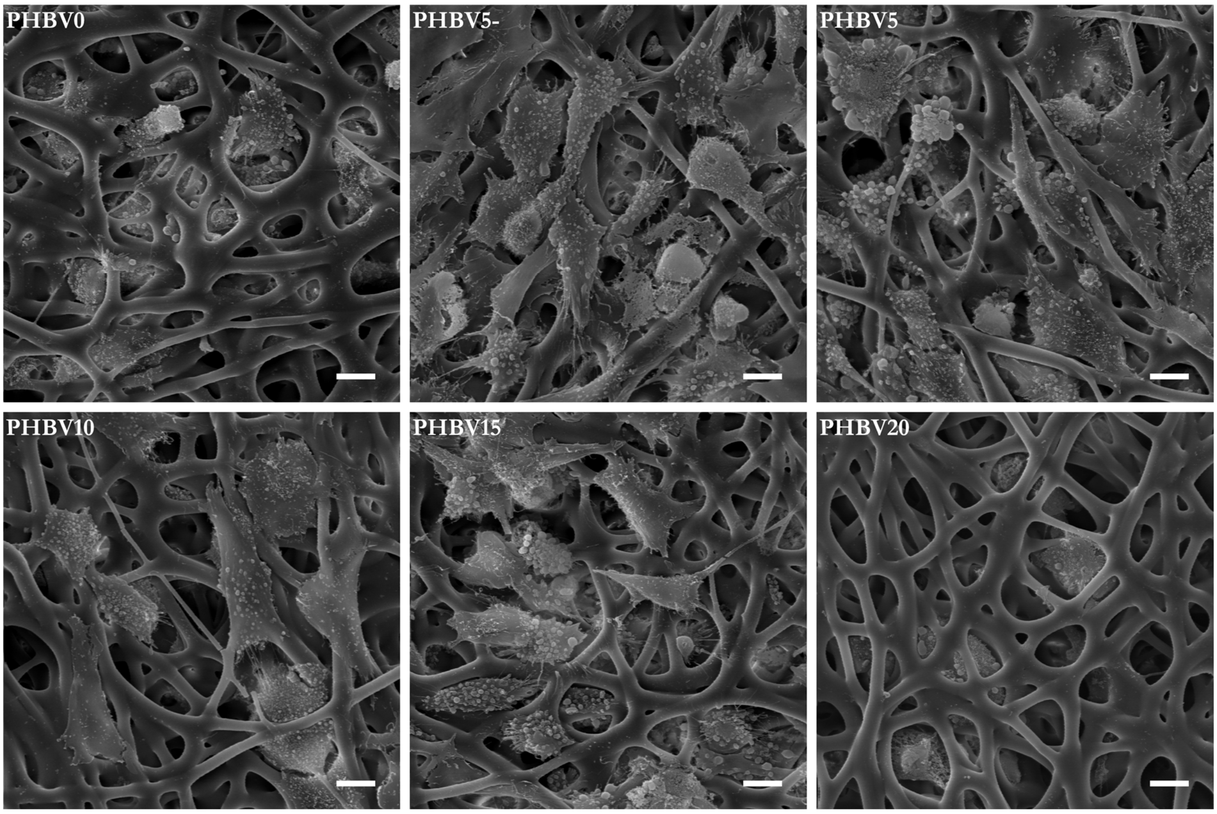

Figure S4. SEM images of SaOS-2 cells cultured for 7 days on the different modified PHBV fibers (scale bars are all 10 μm).
